# Difference in 30-Day Readmission Rates After Laparoscopic Sleeve Gastrectomy Versus Laparoscopic Roux-En-Y Gastric Bypass: a Propensity Score Matched Study Using ACS NSQIP Data (2015–2019)

**DOI:** 10.1007/s11695-022-06446-6

**Published:** 2023-01-28

**Authors:** Md Ashfaq Ahmed, Zhenwei Zhang, Venkataraghavan Ramamoorthy, Anshul Saxena, Muni Rubens, Sandeep Appunni, Peter McGranaghan, Ahmed Hasnain Jalal, Emir Veledar

**Affiliations:** 1 Baptist Health South Florida, Miami, FL USA; 2 Florida International University, Miami, FL USA; 3 Government Medical College, Kozhikode, India; 4 Charité-Universitätsmedizin Berlin, Corporate Member of Freie Universität Berlin and Humboldt Universität Zu Berlin, Berlin, Germany; 5 Department of Electrical Engineering, The University of Texas Permian Basin, Odessa, TX USA

**Keywords:** Bariatric surgery, NSQIP, Propensity score matching, Stomach

## Abstract

**Purpose:**

There are very few studies that have compared the short-term outcomes of laparoscopic Roux-en-Y gastric bypass (LRYGB) and laparoscopic sleeve gastrectomy (LSG). Among short-term outcomes, hospital readmission after these procedures is an area for quality enhancement and cost reduction. In this study, we compared 30-day readmission rates after LSG and LRYGB through analyzing a nationalized dataset. In addition, we identified the reasons of readmission.

**Materials and Methods:**

The current study was a retrospective analysis of data from National Surgical Quality Improvement Program (NSQIP) All adult patients, ≥ 18 years of age and who had LSG or LRYGB during 2014 to 2019 were included. Current Procedural Terminology (CPT) codes were used to identify the procedures. Multivariate logistic regressions were used to calculate propensity score adjusted odds ratios (ORs) for all cause 30-day re-admissions.

**Results:**

There were 109,900 patients who underwent laparoscopic bariatric surgeries (67.5% LSG and 32.5% LRYGB). Readmissions were reported in 4168 (3.8%) of the patients and were more common among RYGB recipients compared to LSG (5.6% versus 2.9%, *P* < 0.001). The odds of 30-day readmissions were significantly higher among LRYGB group compared to LSG group (AOR, 2.20; 95% CI; 1.83, 2.64). In addition, variables such as age, chronic obstructive pulmonary disease, hypertension, bleeding disorders, blood urea nitrogen, SGOT, alkaline phosphatase, hematocrit, and operation time were significantly predicting readmission rates.

**Conclusions:**

Readmission rates were significantly higher among those receiving LRYGB, compared to LSG. Readmission was also affected by many patient factors. The factors could help patients and providers to make informed decisions for selecting appropriate procedures.

**Graphical Abstract:**

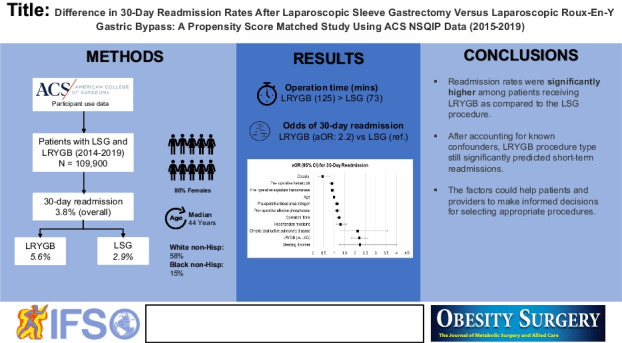

## Introduction

The World Health Organization considers obesity to be a major public health challenge [1]. According to global estimates, there are 400 million obese adults and 1.6 billion overweight adults. About 300,000 deaths occur yearly because of complications of obesity such as heart disease, diabetes, hypertension, and cancer [2]. Thus, obesity is responsible for a significant proportion of preventable deaths. According to recent estimates, one-third of American adults are overweight or obese. Annually, a substantial portion of (10%) of healthcare expenditures in the USA are spent on the treatment of obesity or associated surgical and medical complications [3].

There has been limited success in improving obesity-related comorbidities through non-operative measures such as increased physical activity, dietary modification, and behavioral interventions [4]. Bariatric surgery remains the only effective and sustainable solution to permanently combat morbid obesity and its related complications [5]. Bariatric procedures decrease mortality by as much as 35 to 89% by significantly improving or completely resolving obesity associated chronic comorbidities such as hypertension, diabetes, obstructive sleep apnea, and several more of the cardiovascular risks [6]. A substantial number of patients also report improved quality of life and better psychosocial outcomes as a result of these interventions [3].

Bariatric surgeries have improved significantly over the past decades. Conventional bariatric procedures such as vertical and horizontal gastroplasty and jejunoileal bypass are not performed currently due to several associated complications [7]. For the same reasons, majority of the bariatric surgical centers have also discontinued biliopancreatic diversion with duodenal switch [8]. In the late 1960s, Mason and colleagues combined the aforementioned techniques to develop a procedure that subsequently underwent many modifications and evolved into the current laparoscopic Roux-en-Y gastric bypass (LRYGB) technique [9]. In RYGB technique, a small pouch is created within the stomach so that food intake in minimized. A part of the small intestine is attached to this pouch to allow food to bypass the primary digestion assisting portions of the stomach and small intestine. Many practitioners consider LRYGB to be the gold standard of bariatric surgery since it has proven to be so successful. Until recently, the LRYGB was one of the most commonly performed bariatric procedures in the USA due to the high degree of safety and excellent long-term results. However, laparoscopic sleeve gastrectomy (LSG) which is a relatively new bariatric procedure is gaining support over LRYGB in the recent years [10]. Although initially recommended through a 2-stage approach, LSG has later modified as a stand-alone one step procedure for the treatment of morbid obesity [11]. In LSG, the stomach [12, 13] is reduced in capacity by removing two-thirds of the organ and suturing the remaining part using a stapling device. Several studies have supported that LSG showed similar weight loss and reduction in obesity-related comorbidities to LRYGB [14, 15]. Some studies have also shown that LSG has similar or even lower rates of postoperative complications and 30-day risk-adjusted mortality compared to LRYGB [14, 16, 17]. Due to these reasons, LSG is becoming more common over LRYGB over the past few years [10, 18].

Although LSG is associated with fewer technical challenges compared to LRYGB [14], very few large scale studies have compared the short-term outcomes between the two procedures. Hospital readmissions after surgery constitutes an area that could be utilized for quality enhancement and cost reduction [19, 20]. Identification of risk factors for readmission may predict patients who are candidates for targeted interventions. Majority of the readmissions after bariatric surgeries are potentially preventable as they are often symptom-related. Consequently, research on this topic has become one of the most important focus area in the field [21]. Current consensus suggests that readmission rates are 1.1 to 9.0% for LRYGB and 0.7–5.4% for laparoscopic LSG [16, 22–25]. Nevertheless, LSG being a relatively new procedure, readmission rates and their reasons could be explored further. The aim of this study was to compare the 30-day readmission rates after LSG and LRYGB using a large, validated nationwide dataset. This study also intended to identify the reasons of readmissions after these two bariatric procedures.

## Methods

### Data Source

The current study was a retrospective analysis of American College of Surgeons National Surgical Quality Improvement Program (ACS NSQIP) database collected during the years 2014 to 2019. All patients who underwent LSG or LRYGB during this period and who were ≥ 18 years of age were selected from the NSQIP database. The NSQIP includes data from greater than 400 surgical centers within the USA. This includes urban academic hospitals to small community practice centers. As a third party collecting independent data from each center, the NSQIP has provided validated and reliable measurements of various health quality metrics for research purposes [26].

### Study Population and Variables

Current Procedural Terminology (CPT) code 43,775 was used to identify LSG, while codes 43,644 and 43,645 were used to identify LRYGB from the NSQIP Participant User Files [7]. Patient characteristics were described using age, sex, race, BMI, operation year, ASA class category, smoking, prior comorbidities (obesity, diabetes, chronic obstructive pulmonary disease [COPD], congestive heart failure, hypertension, renal failure, dialysis, and bleeding disorder), preoperative lab variables (sodium, blood urea nitrogen, albumin, creatinine, bilirubin, serum glutamic-oxaloacetate transaminase [SGOT], alkaline phosphatase, white blood cell, hematocrit, platelet, and partial thromboplastin time [PTT]), and operation time.

### Statistical Analysis

Descriptive statistics and outcomes were analyzed in the unmatched cohorts using *χ*^2^ test for categorical variables and Kruskal–Wallis test for continuous variables. To adjust for baseline differences between cohorts, a propensity score algorithm was used to match LSG patients to LRYGB patients in a 1:1 ratio. Propensity score matching (PSM) is a well-validated statistical technique that creates comparable groups and allows for accurate assessment of treatment effects [27]. Patients were matched for race, gender, comorbidities, and laboratory values. Following PSM a random sample of 60,000 cases by proportional allocation was selected. After matching, demographics, presurgical comorbidities, and outcomes of interest were compared using *χ*^2^ (Fischer’s exact when appropriate) and Kruskal–Wallis test for categorical and continuous variables, respectively. Multivariate logistic regression analysis was used to generate propensity score adjusted odds ratios (ORs) for all cause 30-day readmissions. Backward selection was applied to construct the logistic model. All demographics, comorbidities, and operative factors used to generate the propensity score were screened for inclusion in each model. Variables with *P* < 0.05 and number of events ≥ 10 was included in the model. In one multivariate logistic regression analysis, continuous variables were standardized using interquartile range in SAS using proc stdize procedure. This was done to remove the effects of differing scales of these variables [28]. All statistical analyses were performed using SAS 9.4 (Cary, NC).

## Results

There were 109,900 patients who underwent laparoscopic bariatric surgery (67.5% LSG and 32.5% LRYGB) between 2014 and 2019. The majority of the patients were females (80.1%). Median (interquartile range [IQR]) age was 44.0 (35.0, 53.0) years and median (IQR) BMI was 43.9 (39.9, 49.3) kg/m^2^. Operation time was significantly higher in LRYGB patients compared to LSG patients (125.0 min versus 73.0 min, *P* < 0.001). Readmission was reported in 4168 (3.8%) patients. Readmissions were more common among those receiving RYGB, compared to LSG (5.6% versus 2.9%, *P* < 0.001). Baseline characteristics based on procedure performed and readmission rates are shown in Table 1.

**Table 1:** Demographic and clinical characteristics of patients receiving LSG and LRYGB

Variables	Overall *n* = 109,900	LSG *n* = 74,140	LRYGB *n* = 35,760	*P* value
Age, median (Q1, Q3)	44.0 (35.0,53.0)	44.0 (35.0,53.0)	45.0 (36.0,53.0)	< 0.001
Race, *n* (%)				
White non-Hispanic	63,384 (57.7)	42,670 (57.6)	20,714 (57.9)	< 0.001
Black non-Hispanic	16,258 (14.8)	12,268 (16.5)	3990 (11.2)	
Hispanic	12,793 (11.6)	8704 (11.7)	4089 (11.4)	
Others	17,465 (15.9)	10,498 (14.2)	6967 (19.5)	
Sex, *n* (%)				
Male	21,919 (19.9)	15,345 (20.7)	6574 (18.4)	< 0.001
Female	87,981 (80.1)	58,795 (79.3)	29,186 (81.6)	
Obesity, *n* (%)				
No	1048 (1.0)	529 (0.7)	519 (1.5)	< 0.001
Yes	108,852 (99.0)	73,611 (99.3)	35,241 (98.5)	
BMI, median (IQR)	43.9 (39.9,49.3)	43.6 (39.9,49.0)	44.3 (40.1,49.9)	< 0.001
Operation year, *n* (%)				
2015	24,828 (22.6)	16,174 (21.8)	8654 (24.2)	< 0.001
2016	26,325 (24.0)	17,983 (24.3)	8342 (23.3)	
2017	26,792 (24.4)	18,598 (25.1)	8194 (22.9)	
2018	19,467 (17.7)	13,271 (17.9)	6196 (17.3)	
2019	12,488 (11.4)	8114 (10.9)	4374 (12.2)	
ASA class, *n* (%)				
0	27,542 (25.1)	20,167 (27.2)	7375 (20.6)	< 0.001
1	82,358 (74.9)	53,973 (72.8)	28,385 (79.4)	
Diabetes, *n* (%)				
Insulin	9099 (8.3)	4792 (6.5)	4307 (12.0)	< 0.001
No	81,753 (74.4)	57,249 (77.2)	24,504 (68.5)	
Non-insulin	19,048 (17.3)	12,099 (16.3)	6949 (19.4)	
Smoke, *n* (%)				
No	100,528 (91.5)	67,552 (91.1)	32,976 (92.2)	< 0.001
Yes	9372 (8.5)	6588 (8.9)	2784 (7.8)	
COPD, *n* (%)				
No	108,048 (98.3)	72,969 (98.4)	35,079 (98.1)	< 0.001
Yes	1852 (1.7)	1171 (1.6)	681 (1.9)	
Congestive heart failure (CHF), *n* (%)				
No	109,466 (99.6)	73,821 (99.6)	35,645 (99.7)	0.008
Yes	434 (0.4)	319 (0.4)	115 (0.3)	
Hypertension medicine, *n* (%)				
No	59,152 (53.8)	40,684 (54.9)	18,468 (51.6)	< 0.001
Yes	50,748 (46.2)	33,456 (45.1)	17,292 (48.4)	
Renal failure, *n* (%)				
No	109,862 (100.0)	74,108 (100.0)	35,754 (100.0)	0.042
Yes	38 (0.0)	32 (0.0)	6 (0.0)	
Dialysis, *n* (%)				
No	109,481 (99.6)	73,787 (99.5)	35,694 (99.8)	< 0.001
Yes	419 (0.4)	353 (0.5)	66 (0.2)	
Steroid, *n* (%)				
No	107,747 (98.0)	72,565 (97.9)	35,182 (98.4)	< 0.001
Yes	2153 (2.0)	1575 (2.1)	578 (1.6)	
Bleeding disorder, *n* (%)				
No	108,805 (99.0)	73,403 (99.0)	35,402 (99.0)	0.938
Yes	1095 (1.0)	737 (1.0)	358 (1.0)	
Pre-operative variables				
Sodium, median (IQR)	139.0 (138.0,141.0)	139.0 (138.0,141.0)	139.0 (138.0,141.0)	< 0.001
Blood urea nitrogen, median (IQR)	14.0 (11.0,18.0)	14.0 (11.0,18.0)	14.0 (11.0,18.0)	0.002
Creatinine, median (IQR)	0.8 (0.7,0.9)	0.8 (0.7,0.9)	0.8 (0.7,0.9)	0.416
Albumin, median (IQR)	4.1 (3.9,4.3)	4.1 (3.9,4.4)	4.1 (3.8,4.3)	< 0.001
Bilirubin, median (IQR)	0.4 (0.3,0.6)	0.4 (0.3,0.6)	0.4 (0.3,0.6)	0.053
SGOT, median (IQR)	21.0 (16.0,28.0)	21.0 (16.0,28.0)	21.0 (16.0,28.0)	0.089
Alkaline phosphatase, median (IQR)	77.0 (64.0,92.0)	76.0 (64.0,92.0)	77.0 (64.0,93.0)	< 0.001
White blood cell, median (IQR)	7.8 (6.5,9.3)	7.8 (6.4,9.3)	7.9 (6.6,9.4)	< 0.001
Hematocrit, median (IQR)	40.8 (38.6,43.0)	40.8 (38.5,43.0)	40.9 (38.7,43.0)	0.083
Platelet count, median (IQR)	271.0 (230.0,317.0)	271.0 (230.0,317.0)	271.0 (230.0,316.0)	0.453
PTT, median (IQR)	29.3 (26.8,32.0)	29.5 (27.0,32.1)	29.0 (26.2,32.0)	< 0.001
Operation time, median (IQR)	87.0 (62.0,122.0)	73.0 (55.0,97.0)	125.0 (96.0,163.0)	< 0.001
Total hospital length of stay, median (IQR)	2.0 (1.0,2.0)	2.0 (1.0,2.0)	2.0 (1.0,2.0)	< 0.001
30-day readmission, *n* (%)				
No	105,732 (96.2)	71,985 (97.1)	33,747 (94.4)	< 0.001
Yes	4168 (3.8)	2155 (2.9)	2013 (5.6)	

Abbreviations: *BMI*, body mass index; *ASA*, American Society of Anesthesia; *COPD*, chronic obstructive pulmonary disease

 LRYGB comorbidities such as insulin-dependent diabetes (12.0% versus 6.5%, *P* < 0.01), non-insulin dependent diabetes (19.4% versus 16.3%, *P* < 0.01), COPD (1.9% versus 1.6%, *P* < 0.01), and hypertension (48.4% versus 45.1%, *P* < 0.01) were higher among those who underwent LRYBG, while smoking history (8.9% versus 7.8%, *P* < 0.01), congestive heart failure (0.4% versus 0.3%, *P* < 0.01), dialysis (0.5% versus 0.2%, *P* < 0.01), and chronic steroid use (2.1% versus 1.6%, *P* < 0.01) were higher among those who underwent LSG. The distribution of comorbidities between LSG and LRYGB groups are shown in Table 1. To adjust for baseline differences between cohorts, a propensity score algorithm was used to match LSG patients to LRYGB patients in a 1:1 ratio. After propensity score matching, there was no significant difference in COPD, congestive heart failure, and bleeding disorder between LSG and LRYGB patients. However, LRYGB patients still had higher rates of diabetes (insulin-dependent 12.1% versus 6.5%, *P* < 0.01; non-insulin dependent 19.4% versus 16.4%, *P* < 0.01) and hypertension (48.3% versus 45.5%, *P* < 0.01). In the matched cohort, the LSG group compared to the LRYGB group had a higher dialysis (0.5% versus 0.2%, *P* < 0.01) and chronic steroid use (2.1% versus 1.6%, *P* < 0.01). Table 2 shows demographic data and baseline characteristics of the patients after propensity score matching.

**Table 2:** Demographic data and baseline characteristics of the patients after propensity score matching

Variables	Overall *n* = 60,000	SG *n* = 30,000	RYGB *n* = 30,000	*P* value
Age, median (IQR)	45.0 (36.0, 53.0)	45.0 (36.0, 53.0)	45.0 (36.0, 53.0)	0.798
Race, *n* (%)				
White non-Hispanic	34,712 (57.9)	17,320 (57.7)	17,392 (58.0)	0.698
Black non-Hispanic	6665 (11.1)	3306 (11.0)	3359 (11.2)	
Hispanic	6810 (11.3)	3423 (11.4)	3387 (11.3)	
Others	11,813 (19.7)	5951 (19.8)	5862 (19.5)	
Gender, *n* (%)				
Male	11,004 (18.3)	5500 (18.3)	5504 (18.3)	0.975
Female	48,996 (81.7)	24,500 (81.7)	24,496 (81.7)	
Obesity, *n* (%)				
No	740 (1.2)	336 (1.1)	404 (1.3)	0.013
Yes	59,260 (98.8)	29,664 (98.9)	29,596 (98.7)	
BMI, median (IQR)	43.9 (39.9, 49.3)	43.4 (39.6, 48.7)	44.3 (40.2, 49.9)	< 0.001
ASA class, *n* (%)				
0	14,514 (24.2)	8350 (27.8)	6164 (20.5)	< 0.001
1	45,486 (75.8)	21,650 (72.2)	23,836 (79.5)	
Diabetes, *n* (%)				
Insulin	5582 (9.3)	1958 (6.5)	3624 (12.1)	< 0.001
No	43,679 (72.8)	23,122 (77.1)	20,557 (68.5)	
Non-insulin	10,739 (17.9)	4920 (16.4)	5819 (19.4)	
Smoke, *n* (%)				
No	54,939 (91.6)	27,260 (90.9)	27,679 (92.3)	< 0.001
Yes	5061 (8.4)	2740 (9.1)	2321 (7.7)	
COPD, *n* (%)				
No	58,937 (98.2)	29,494 (98.3)	29,443 (98.1)	0.122
Yes	1063 (1.8)	506 (1.7)	557 (1.9)	
Congestive heart failure, *n* (%)				
No	59,775 (99.6)	29,874 (99.6)	29,901 (99.7)	0.082
Yes	225 (0.4)	126 (0.4)	99 (0.3)	
Hypertension medicine, *n* (%)				
No	31,881 (53.1)	16,364 (54.5)	15,517 (51.7)	< 0.001
Yes	28,119 (46.9)	13,636 (45.5)	14,483 (48.3)	
Renal failure, *n* (%)				
No	59,980 (100.0)	29,985 (100.0)	29,995 (100.0)	0.044
Yes	20 (0.0)	15 (0.1)	5 (0.0)	
Dialysis, *n* (%)				
No	59,800 (99.7)	29,847 (99.5)	29,953 (99.8)	< 0.001
Yes	200 (0.3)	153 (0.5)	47 (0.2)	
Steroid, *n* (%)				
No	58,889 (98.1)	29,375 (97.9)	29,514 (98.4)	< 0.001
Yes	1111 (1.9)	625 (2.1)	486 (1.6)	
Bleeding disorder, *n* (%)				
No	59,408 (99.0)	29,700 (99.0)	29,708 (99.0)	0.772
Yes	592 (1.0)	300 (1.0)	292 (1.0)	
Pre-operative variables				
Sodium, median (IQR)	139.0 (138.0, 141.0)	139.0 (138.0, 141.0)	139.0 (138.0, 141.0)	< 0.001
Blood urea nitrogen, median (IQR)	14.0 (11.0, 18.0)	14.0 (11.0, 18.0)	14.0 (11.0, 18.0)	0.173
Creatinine, median (IQR)	0.8 (0.7, 0.9)	0.8 (0.7, 0.9)	0.8 (0.7, 0.9)	0.022
Albumin, median (IQR)	4.1 (3.8, 4.3)	4.1 (3.9, 4.4)	4.1 (3.8, 4.3)	< 0.001
Bilirubin, median (IQR)	0.4 (0.3, 0.6)	0.4 (0.3, 0.6)	0.4 (0.3, 0.6)	0.014
SGOT, median (IQR)	21.0 (16.0, 28.0)	21.0 (16.0, 28.0)	21.0 (16.0, 28.0)	0.004
Alkaline phosphatase, median (IQR)	77.0 (64.0, 93.0)	77.0 (64.0, 93.0)	77.0 (64.0, 93.0)	0.072
White blood cell, median (IQR)	7.8 (6.5, 9.4)	7.8 (6.5, 9.3)	7.9 (6.6, 9.4)	< 0.001
Hematocrit, median (IQR)	40.8 (38.6, 43.0)	40.8 (38.6, 43.0)	40.9 (38.7, 43.0)	0.775
Platelet count, median (IQR)	271.0 (230.0, 317.0)	271.0 (230.0, 317.0)	271.0 (230.0, 317.0)	0.920
PTT, median (IQR)	29.0 (26.4, 32.0)	29.2 (26.5, 32.1)	29.0 (26.2, 32.0)	< 0.001
Operation time, median (IQR)	97.0 (69.0, 135.0)	74.0 (55.0, 98.0)	125.0 (96.0, 163.0)	< 0.001
Total hospital length of stay, median (IQR)	2.0 (1.0, 2.0)	2.0 (1.0, 2.0)	2.0 (1.0, 2.0)	< 0.001
30-day readmission, *n* (%)				
No	57,479 (95.8)	29,125 (97.1)	28,354 (94.5)	< 0.001
Yes	2521 (4.2)	875 (2.9)	1646 (5.5)	

After accounting for covariates, the LRYGB group showed significantly higher odds of 30-day readmission (AOR, 1.81; 95% CI: 1.48, 2.21), compared to the LSG group, without standardization (Table 3). This association remained significant after data standardization procedure (AOR, 2.20; 95% CI: 1.83, 2.64) (Table 4). In addition, variables such as age, chronic obstructive pulmonary disease, hypertension, bleeding disorder, blood urea nitrogen, SGOT, alkaline phosphatase, hematocrit, and operation time were significant predictors of readmission.

**Table 3:** Factors associated with 30-day readmission following bariatric surgery estimated by conditional logistic regression without standardization

Variables	AOR (95% CI)
Age	0.99 (0.98–0.99)
Obesity	0.44 (0.25–0.77)
Chronic obstructive pulmonary disease	2.12 (1.26–3.56)
Hypertension medicine	1.30 (1.07–1.59)
Bleeding disorder	2.23 (1.24–4.02)
Pre-operative blood urea nitrogen	1.02 (1.01–1.03)
Pre-operative aspartate transaminase	0.99 (0.99–1.00)
Pre-operative alkaline phosphatase	1.01 (1.00–1.01)
Pre-operative hematocrit	0.97 (0.95–1.00)
Operation time	1.00 (1.00–1.01)
Procedure	
LSG	Reference
LRYGB	1.81 (1.48–2.21)

**Table 4:** Factors associated with 30-day readmission following bariatric surgery estimated by conditional logistic regression without standardization

Variables	AOR (95% CI)
Age	0.99 (0.98–0.99)
Obesity	0.45 (0.26–0.79)
Chronic obstructive pulmonary disease	2.12 (1.26–3.56)
Hypertension medicine	1.30 (1.07–1.59)
Bleeding disorder	2.23 (1.24–4.01)
Pre-operative blood urea nitrogen	1.13 (1.06–1.22)
Pre-operative aspartate transaminase	0.90 (0.84–0.98)
Pre-operative alkaline phosphatase	1.14 (1.05–1.24)
Pre-operative hematocrit	0.89 (0.80–0.98)
Operation time	1.23 (1.13–1.33)
Procedure	
LSG	Reference
LRYGB	2.2 (1.83–2.64)

## Discussion

There has been exponential increase in the prevalence of all categories of obesity, and consequently, the rates of bariatric surgeries have also increased [5]. Recent research has supported that bariatric surgeries are safe and effective procedures for achieving and sustaining long-term weight loss [6, 29]. These procedures have significantly decreased the comorbidities associated with overweight and obesity and resulted in significant savings in healthcare expenditures [30, 31]. Since bariatric surgeries have been accepted as safe and effective, recent studies have started comparing the benefits versus adverse effects of existing bariatric procedures [32, 33]. Therefore, we sought to understand the short-term safety and outcomes of the two commonly performed stapling bariatric procedures in the USA. Our study could help practitioners to counsel patients preoperatively and make appropriate selections.

Selecting the ideal bariatric procedure should be based on personalized and patient centered factors such as age, comorbidities, BMI, and individual choices as well as provider related factors such as level of expertise and availability of resources. Patients should also be informed about the risk involved with bariatric procedures and complications and preoperative procedures. While making informed decisions for bariatric surgeries, both patients and provider should also consider other outcome measures such as hospital length of stay and readmission rates, as well as financial factors such as affordability, insurance coverage, and reimbursements. Most of the previous studies which looked for outcomes after bariatric surgeries have emphasized on factors associated with risk for reoperation, morbidity, and mortality. Although a few studies have used large-scale database such as NSQIP, similar studies on readmission in different settings are scarce [34].

Given the significant number of potential confounders, understanding the independent impact of any bariatric procedure (in our case, LSG and LRYGB) could be challenging. To overcome this issue, we used a strict propensity score match, which was able to adequately control for confounding variables. Our data showed that even after propensity score matching, the rates of 30-day readmissions were significantly higher after LRYGB, compared to LSG. This suggests that LRYGB is independently associated with increased readmission rates, while patients undergoing LSG are less likely to be readmitted. This finding is consistent with a recent study of New York statewide data showing a significantly higher readmission rate of 6.14% after LRYGB, compared to 4.33% after LSG [21]. Using NSQIP data, Young et al. also found that readmissions were more common after LRYGB than LSG (6.08% versus 4.05%, *P* < 0.01) [16]. However, in this study, readmission data were available only for 2011 ACS NSQIP containing a much smaller sample size. In addition, two studies have reported higher readmission rates after LRYGB compared to LSG, though non-significant [35, 36]. However, these non-significant results could be due to smaller number of LSG procedures in these studies. One notable study employed Metabolic and Bariatric Surgery Accreditation and Quality Improvement Program data from 2015 to 2018 to determine risk factors for readmission after bariatric surgery [37]. However, in contrast to our study, the authors did not perform any propensity score matching to adequately control for confounding variables. Other factors significantly associated with unplanned readmissions included younger age, COPD, antihypertensive medication use, bleeding disorders, pre-operative blood urea nitrogen, SGOT, alkaline phosphatase, hematocrit, and operative time. This is similar to many previous studies attempting to predict readmission after bariatric and complex intestinal surgeries [22, 38].

Our retrospective analysis demonstrated that patients undergoing LRYGB have a higher rate of readmission compared to those who underwent LSG, even after propensity score matching. One possible reason could be that the two groups were differing with respect to unmeasured confounders. This may have resulted in apparently higher rates of readmission for those who had higher comorbidity burden at baseline, for example, tobacco user with diabetes who underwent LRYGB versus patients with obesity with mild hypertension who underwent LSG. Notably, the LRYGB group had greater prevalence of insulin-dependent diabetes (12.1% versus 6.5%, *P* < 0.01) and were more at risk for cardiac complications as indicated by their higher rates of antihypertensive medication use (48.3% versus 45.5%, *P* < 0.01). These results are consistent with those reported by Spaniolas et al., who showed a higher prevalence of diabetes among LRYGB patients, compared to LSG (56.6% versus 43.2%, *P* < 0.01) [17].

### Strengths and Limitations

We used a large-scale database such as NSQIP which has strengthened our study. We could analyze data from a large sample of bariatric surgery patients, and this greatly improved the accuracy of our estimates. This database includes both community and academic hospitals across the USA, thereby increasing the generalizability of our findings. In addition, a large number of perioperative variables were available for analysis. Thus, the NSQIP database provides clear benefits of power and heterogeneity of practice, type, and volume, offering one of the best possible representations of bariatric surgery at the national level.

Our study has some limitations. We conducted a retrospective analysis of NSQIP database. Our data included only those hospitals that participated in NSQIP. This could have resulted in some selection bias. NSQIP is an administrative database and not bariatric specific. Therefore, only those variables that were collected by NSQIP were available for analysis. NSQIP does not have data on providers or volume of procedures occurring in the hospitals. This could have affected our findings on readmissions as readmission is associated with factors such as bariatric accreditation status and volume of procedures [39, 40]. Important co-morbidities such as obstructive sleep apnea, history of DVT, and complications such as bowel obstruction, gastrointestinal hemorrhage, and anastomotic stricture are not recorded in the database [41]. These unmeasured confounders could have influenced the comparison of LSG versus RYGB. The ACS-NSQIP dataset only collects data for the first 30 postoperative days and does not account for readmissions beyond the 30-day postoperative period. This is because “readmission,” as defined by the NSQIP, includes patients who are readmitted within 30 days of their operation, while the Centers for Medicare and Medicaid (CMS) use 30 days from the date of hospital discharge [42]. In a study that retrospectively looked for one year follow-up after bariatric procedure, only 36% of readmissions occurred within 30 days after the procedure [43]. Therefore, we could not compare postoperative readmissions beyond 30 days post-SG and post-RYGB. Although ACS-NSQIP attempts to capture readmission data via patient communication and review of medical records, it is possible that some readmissions are uncaptured. Lastly, ACS-NSQIP does not have data on ED visits. This could have prevented the capture of information from patients who would have postoperatively visited EDs but were not readmitted. Although not the objective of this study, availability of such data could have helped to understand whether differences existed between bariatric procedures with respect to ED utilization, which did not result in hospitalizations.

## Conclusion

Using propensity score matched analysis we found that 30-day readmission were significantly higher among those receiving LRYGB, compared to LSG. In addition to the procedure type, readmission is also affected by many patient factors. These factors should be discussed by both patients and providers while selecting appropriate procedures. Our findings should be confirmed by future large-scale experimental studies.

## Data Availability

Data is available from the National Surgical Quality Improvement Program (NSQIP) database upon request.
